# Parental Attitudes toward Gifted Students and Gifted Education: Attitude Profiles and Predictors

**DOI:** 10.3390/jintelligence12050048

**Published:** 2024-04-29

**Authors:** Jae Yup Jung, Jihyun Lee

**Affiliations:** School of Education, The University of New South Wales, Sydney 2052, Australia; jihyun.lee@unsw.edu.au

**Keywords:** parents, attitude, giftedness, gifted students, gifted education, predictors

## Abstract

In this study, an investigation was conducted into the types of attitudes that parents may have of gifted students and gifted education, and the predictors of these attitudes. Using data collected from 331 parents of students enrolled in a Christian faith-based school system in one of the eight states/territories of Australia, multiple analyses, including exploratory factor analysis and latent profile analysis, were performed. The results revealed three subgroups of parents, each representing distinct attitude profiles (i.e., “strong”, “moderate” and “weak” supporters of gifted students and gifted education). Furthermore, we found nine variables to be potential predictors of parent attitudes, including perceptions of the giftedness of one’s child, and the anticipated socio-emotional and academic impacts of giftedness and gifted education. Some of the important contributions of the study to the research literature included the distinction made by parents between attitudes toward gifted education adaptations and attitudes toward special gifted education settings, and the comparatively large number of parents who are moderately (rather than strongly or weakly) supportive of gifted students and gifted education.

## 1. Introduction

The focus of this study was on the *attitudes* of parents toward gifted students and gifted education. Multiple definitions of attitude exist. They range from simple definitions such as those that relate it to one’s “likes and dislikes” (Bem 1970, p. 14) to more developed conceptualizations that recognize it to be a “psychological tendency that is expressed by evaluating a particular entity with some degree of favor or disfavor” (Eagly and Chaiken 1993, p. 1). Nevertheless, most definitions appear to consider it to be a relatively enduring and evaluative construct that may motivate a response to a particular object (Albarracin et al. 2018; Oskamp and Schultz 2005). One of the most recognized theoretical perspectives on attitudes is the *tricomponent view*, which considers attitude to be a single entity that nevertheless comprises three highly correlated components—a cognitive component (i.e., one’s ideas and beliefs toward an object), an affective component (i.e., one’s feelings and emotions toward an object), and a behavioral component (i.e., one’s tendencies for action with respect to an object; Fabringar et al. 2018; Oskamp and Schultz 2005). This theoretical perspective has been commonly adopted in the fields of social psychology and special education.

Within the field of gifted education, guided by Gagné’s (2009) Differentiated Model of Giftedness and Talent (DMGT) in Australia (Thraves 2024), attitudes have largely been investigated using a perspective that differs from the tricomponent view, but nevertheless aligns with the dichotomous “like vs. dislike” or “favorable vs. disfavorable” conceptualization incorporated into the commonly adopted definitions of attitude. That is, studies that have investigated general attitudes toward gifted students and gifted education have typically done so in terms of whether one may be “supportive” or have non-supportive “perceptions of elitism” (Bégin and Gagné 1995; Jung 2014; McCoach and Siegle 2007; Mullen and Jung 2019; Palacios Gonzalez and Jung 2021; Wirthwein et al. 2019). Supportive attitudes are consistent with the *harmony hypothesis* which proposes an overall positive view of giftedness and gifted education that encompasses a high level of ability, educational/career success and sound socio-emotional adjustment (Baudson and Preckel 2013, 2016), along with the recognition of the importance of achievement and creative productivity in promoting the “knowledge” economy and a country’s international competitiveness (Lassig 2009).

On the other hand, perceptions of elitism may be associated with ambivalent, apathetic, and otherwise negative attitudes that may reflect an egalitarian worldview, a perception that gifted students are already privileged, or common stereotypes about the socio-emotional difficulties of gifted students (Baudson and Preckel 2013, 2016; Callahan 2017). Some proponents of this view argue that gifted education practices such as ability grouping often lead to the segregation of students by race and socio-economic status, reflecting differences in the socio-economic and political capital of their parents (i.e., “elite” parents vs. “non-elite” parents), and leading to unequal educational opportunities, resources and learning environments (Oakes et al. 2014; Roda and Wells 2013; Wells and Serna 1996). Oakes et al. (2012) have noted that some have gone as far as to suggest that gifted education practices are “a masquerade sometimes for institutional racism and classism” (p. 303).

The existence of these different perspectives is reflected in the lack of consensus on the topic in multiple studies to date that have collectively identified substantial variation in attitudes, that range from support to rejection of gifted students and gifted education (Bégin and Gagné 1994, 1995; Wirthwein et al. 2019). One possible explanation for the variation in attitudes is the differing interests, priorities and conceptions within and among various stakeholders in the education of gifted students. For example, policymakers may or may not view the value of gifted education in terms of its potential benefit to general education, while academics may or may not see the value of gifted education in terms of its potential for career development (Jung 2019; Shore 2021).

Of note to this study, the majority of past studies on attitudes toward giftedness and gifted education relate to the attitudes of teachers (Jung 2014; McCoach and Siegle 2007; Mullen and Jung 2019; Palacios Gonzalez and Jung 2021; Troxclair 2013). Indeed, multiple scholars have acknowledged the comparative lack of attention to parental attitudes, or to related concepts such as parental perceptions and parental issues related to gifted students and gifted education (Jolly and Matthews 2012; Morawska and Sanders 2009; Wirthwein et al. 2019). In some studies that have investigated parent attitudes, the perspectives of multiple stakeholder groups have been combined. For example, the comprehensive review of the literature by Bégin and Gagné (1994) synthesized the perspectives of parents, teachers, students, psychologists and the general public, and recognized some variables that may represent potentially promising predictors of parental attitudes (i.e., self-perceptions of giftedness, contact with gifted persons, educational attainment, and gender). Similarly, Bégin and Gagné (1995) merged data from parents and teachers, and identified two significant predictors of attitudes toward gifted students and gifted education—socioeconomic status and contact with gifted persons (i.e., oneself, one’s child and others).

Other studies have focused solely on parents. Only a few such studies have incorporated the perspectives of the general parent body, which includes the parents of both gifted and non-gifted students. For example, Makel (2009) recognized that the parents of gifted students tended to have more positive attitudes toward the academic aspects of participation in a gifted program than the parents of non-gifted students, to suggest that the gifted status of one’s child may be an important predictor of parental attitudes. Nevertheless, Colangelo and Kelly (1983) found that the parents of both gifted and non-gifted students may have a desire for their children to participate in gifted programs, to infer that the parents of non-gifted students may also be supportive of gifted programs if they are accessible to their children. In comparison, Wirthwein et al. (2019) found that the parents of gifted students rated their children higher on motivation, intelligence, and general knowledge than parents of non-gifted students, to allow the inference that the parents of gifted students may have more supportive attitudes toward gifted students and gifted education than the parents of non-gifted students.

A relatively larger number of studies have excluded consideration of the parents of non-gifted students, to focus exclusively on the parents of gifted students. Some of these studies may only be broadly related to parental attitudes (e.g., parental perspectives of the needs of gifted students and parental perspectives of the challenges in raising gifted children), which nevertheless allow for inferences about parent attitudes toward gifted students and gifted education. Usually, this group of studies has suggested supportive attitudes (Colangelo and Dettmann 1983; Jolly and Matthews 2012). For example, many of these studies have highlighted the lack of knowledge about gifted students and gifted education, the consequent desire of parents for greater knowledge, and the related efforts to seek this knowledge through self-study and the contact of experts (Colangelo and Dettmann 1983; Foster 2000; Guthrie 2019; Vidergor and Azar Gordon 2015). Other studies have highlighted the many challenges in supporting the socio-emotional development of gifted students, or challenges in supporting gifted students to achieve to their full potential (de Souza Fleith et al. 2024; Guthrie 2019; Morawska and Sanders 2009; Peebles et al. 2023; Wellisch 2021). Of note, the desire to fulfill the potential of their gifted children, and concerns about possible underachievement in the regular classroom, have been cited to be among the reasons why many parents of gifted students have supportive attitudes toward gifted education (Vidergor and Azar Gordon 2015).

Instead of perspectives on overall supportive or non-supportive parental attitudes toward gifted students and gifted education, a number of studies have focused on parental attitudes toward specific elements of gifted education programs. For example, Vidergor and Azar Gordon (2015) identified the features of gifted education programs that parents of gifted students consider to be the most desirable—small student numbers, specially trained teachers, and the development of a sense of belonging, curiosity and motivation to learn in gifted students. Relatedly, Hishinuma and Nishimura (2000) identified high parent ratings (i.e., at least four out of five) across the following components of gifted education programs at a specialized school in the United States—school counseling, behavior management, curriculum/instruction, socio-emotional development, assessment/documentation, and personnel. Other studies have outlined mixed satisfaction with the quality of gifted programs that are offered in schools (Ballam and Sturgess 2019; Hertzog and Bennett 2004). In contrast, Ford and her colleagues (Ford and Grantham 2003; Ford et al. 2019) have noted the common under-representation of certain minority groups in gifted education programs (e.g., African American and Latinx students), which may be a result of deficit thinking (i.e., the holding of negative and stereotypic views about culturally diverse students that result in reduced expectations for these students) by key stakeholders in the education of gifted students.

The generally limited research on the attitudes of parents (of both gifted and non-gifted students) toward gifted students and gifted education, means that only an incomplete picture exists of parental attitudes and the predictors of these attitudes. Possibly, the broader body of research on teacher attitudes toward gifted students and gifted education may provide additional useful insights, particularly with respect to the possible predictors of parental attitudes. Specifically, the research on teacher attitudes has acknowledged the relevance of some predictor variables that have not been recognized in the research on parent attitudes, but may nevertheless be potentially useful:

(a) Rural/urban locality (Azano et al. 2014; Troxclair 2013): As for teachers, parents are likely to be influenced in their attitudes toward gifted students and gifted education by the differences in the educational environment, resources, opportunities, numbers of gifted students, and general community attitudes in rural and urban settings (Jung et al. 2022).
(b) Socio-emotional and academic impacts (Palacios Gonzalez and Jung 2021; Rambo and McCoach 2012; Vialle et al. 2001): Parents, like teachers, are likely to place high priority on the socio-emotional and academic development of their children (Amato and Fowler 2002; Borkowski et al. 2001), which is likely to mean that their attitudes toward gifted students and gifted education may be influenced by their perceptions of the socio-emotional and academic impacts of being gifted, and of gifted education.
(c) School administrative support (Palacios Gonzalez and Jung 2021; Rambo and McCoach 2012): The support of the school leadership for gifted students and gifted education may influence the attitudes of both teachers and parents, because of its possible role in directing and informing the attitudes of the entire school community, and even school culture, with respect to gifted students and gifted education.
(d) Power distance orientation (Jung 2014): Power distance orientation is a cultural orientation variable that refers to the extent to which people (including teachers and parents) consider less powerful members of organizations should accept unequal distributions of power (Hofstede 2001). As part of a well-known model of culture, it addresses a fundamental question faced by societies about how to deal with individual differences in social status, wealth, physical characteristics or *mental* characteristics.

Finally, the research on parental attitudes toward a related group of students (i.e., students with special needs) and the special educational provisions for these students, may provide additional useful insights for this study. In contrast to the research on gifted students and gifted education, this body of research suggests that parents may generally have neutral to supportive attitudes toward special education provisions within regular classrooms and special education settings (de Boer et al. 2010, 2012; Gasteiger-Klicpera et al. 2013). Nevertheless, it also identifies many similar potential predictors of these attitudes, including gender (i.e., female gender may be more predictive of supportive attitudes), educational level/socioeconomic status (i.e., higher levels may be more predictive of supportive attitudes), experience with relevant educational provisions (i.e., experience with inclusive educational provisions may be more predictive of support for inclusive educational provisions), and having a child with special needs (i.e., having a child with special needs may be more predictive of supportive attitudes; de Boer et al. 2010, 2012).

## 2. The Present Study

The purpose of the present study was to investigate the nature or type of attitudes that parents may have toward gifted students and gifted education, along with the predictors of such attitudes. Parental attitudes are critical to study due to the pivotal role they are likely (although not guaranteed) to play in the academic, socio-emotional, and related outcomes of gifted students. Indeed, the most widely adopted model of giftedness in Australia (Thraves 2024), Gagné’s (2009) DMGT, proposes that the exceptional abilities of gifted students (i.e., abilities in the top 10% of age peers) may be transformed into corresponding achievements (i.e., achievements in the top 10% of age peers), through a developmental process that is substantially influenced by intrapersonal factors including motivation and environmental factors including teachers and *parents*. For example, parents may or may not create a home environment that is conducive to the fulfilment of the exceptional potential of gifted students through their parenting practices, routines or the opportunities and resources made available.

As a group, parents have often been neglected in the research on attitudes toward gifted students and gifted education (Guthrie 2019; Wirthwein et al. 2019). This is despite the fact that parents represent one of the key stakeholder groups in the education of gifted students due to their continued presence and involvement in educational decisions, and the substantial impact that their attitudes may have on the attitudes, behaviors, well-being, social adjustment, and academic achievement of gifted students (Campbell and Verna 2007; Colangelo and Dettmann 1983; Freeman 2000). Gaining a clearer and a more complete understanding of the attitudes of parents toward gifted students and gifted education, may allow for the development of appropriate measures (e.g., programs about gifted students/gifted education that are informed by parental attitudes) to support parents to promote optimal outcomes for gifted students.

This study aimed to advance knowledge on parental attitudes toward gifted students and gifted education in several novel ways. First of all, it is the first known study to give simultaneous acknowledgement to the conceptualizations of attitudes in the field of gifted education (i.e., supportive attitudes vs. perceptions of elitism) *and* the fields of social psychology and special education (i.e., cognitive, affective and behavioral attitude), to assess the possible relevance of conceptualizations of attitudes that are widely established outside of gifted education. Relatedly, this study assessed the relevance of potential predictors of these attitudes from predictor variables identified in past research on parent attitudes, along with predictor variables identified to be salient for teachers. Furthermore, in recognition of the possible heterogeneity of parents, which may be related to the existence of different attitudes toward gifted students and gifted education, this study is the first known study to utilize person-centered analytical techniques (i.e., latent profile analysis), rather than the commonly adopted variable-centered analytical techniques that assume cohort homogeneity. Moreover, to address the tendency for past research studies to have an exclusive focus on the parents of gifted students, and to ensure the relevance of the findings to the general parent body, this study incorporated the perspectives of the parents of both gifted and non-gifted students.

The specific research questions that guided the study were as follows:

1. What types of attitudes do parents have toward gifted students and gifted education?
2. What predicts the attitudes of parents toward gifted students and gifted education?

## 3. Materials and Methods

### 3.1. Participants

The study participants comprised the parents of students attending schools that are part of a Christian faith-based school system in Australia. The K–12 school system employs more than 8000 staff teaching more than 50,000 students in more than 100 schools that attract students from a highly diverse range of social, economic, cultural, linguistic, and geographic (i.e., urban and rural) backgrounds. The school system aims to educate and support the “whole person” while instilling Christian values including a commitment to learning, the fulfillment of one’s potential, respect for the dignity of each person, inclusivity, and support for social justice. The gifted education provisions offered at each school, guided by a system-wide gifted education policy that follows Gagné’s (2009) DMGT, include curriculum differentiation, ability grouping, acceleration, and/or extra-curricular activities. Nevertheless, each school appears to prioritize one or more of the gifted education provisions (often curriculum differentiation) over others depending on the nature of its gifted education program, the attitudes of the school leadership, and the perspectives of the gifted coordinator. Furthermore, the gifted education provisions that are offered appear to be dependent on the commitment and training of individual teachers at the school. While many schools in the school system have identification processes, they do not always appear to be rigorous or systematic (e.g., many schools do not have an ongoing identification process using multiple identification instruments, including both traditional and non-traditional instruments).

To recruit participants for this study, all principals of schools in the school system were provided with information about the study by email. Upon receipt of the study information, each principal made the decision on whether their school would participate, and therefore, whether the relevant invitations and information about the study were forwarded to parents at the school. Although 413 parents commenced participation, the surveys returned by 331 parents (whose children attended 35 different schools) were submitted to analysis, as only these surveys had at least 50% of items completed. The participants had a mean age of 45.08 (*SD* = 6.24), and comprised 260 female and 69 male participants, 249 of whom were born in Australia. The major ethnicities of the participants were Anglo-Saxon (*n* = 208), Western European (*n* = 43), Eastern European (*n* = 15), East Asian (*n* = 15), South Asian (*n* = 13), Southeast Asian (*n* = 6), and mixed ethnicity (*n* = 13). More than 70% of the participants (*n* = 238) completed bachelor level study (with 69 having completed more advanced study), and more than 80% of the participants (*n* = 270) lived in an urban area. One hundred and ninety-three of the participants believed that at least one of their children was or may be gifted. Finally, the children of the participants were fairly evenly divided across all of the K–12 grades (i.e., from 5% in Grade 1 to 12% in Grades 7 and 9). 

### 3.2. Survey Instrument

All participants were asked to read an information statement about the study and to consent to participation, before the self-administration of an online survey instrument that was designed to assess their attitudes toward gifted students and gifted education, and the possible predictors of these attitudes. The survey comprised 83 items that are relevant to this study, which were developed from a number of existing scales with strong psychometric properties. As most of the research literature on attitudes toward gifted students and gifted education relate to the attitudes of teachers rather than parents, most of the scales in the survey instrument have their origins in the research literature on teacher attitudes. Therefore, some minor modifications were made, as necessary, to items in some of these scales to suit the study context. Table 1 provides details of the survey instrument used in this study to collect data, including details of the original scales, sample items, and examples of how modifications were made. It is noted that items in the survey instrument were presented randomly to avoid seriation effects.

### 3.3. Analysis

In preparation for analysis of the collected survey data, missing variables that were not unordered-categorical in nature (i.e., comprising 1.5% of data and distributed randomly according to Little’s MCAR test) were imputed using the Expectation-Maximization (EM) algorithm. Thereafter, four items (i.e., one item designed to assess knowledge of giftedness, one item designed to assess academic impact, and two items designed to assess support for gifted programs/provisions) were reverse coded to ensure that all survey items had consistent direction with the other items that were designed to assess each construct. Furthermore, all six items designed to assess perceptions of elitism and socio-emotional impact were reverse coded to ensure that high scores on the scales of these two variables were indicative of a positive attitude (i.e., perceptions of non-elitism) or impact (i.e., positive socio-emotional impact).

#### 3.3.1. Factor Analysis

As a first step, factor analysis was conducted on the survey data to identify an underlying structure in the data comprising groups of highly inter-correlated items, which each assess a unique construct related to parent attitudes toward gifted students/gifted education, or their predictors. Given that the measures employed in this study were based on those used in previous studies, confirmatory factor analysis (i.e., factor analysis that sets a priori constraints on the specific factors to be extracted and the items that assess each factor) was performed first. The survey items that were submitted to confirmatory factor analysis included all items designed to assess the five attitude factors of interest (i.e., support for special gifted programs/provisions, perceptions of non-elitism, cognitive attitude, affective attitude, and behavioral attitude), along with all items that were designed to assess the seven non-sociodemographic predictors of these attitudes (i.e., knowledge of giftedness, contact with gifted persons, self-perceptions of giftedness, power distance orientation, school administrative support, socio-emotional impact, and academic impact). After the removal of 16 items with low communality (i.e., r-square < .30; one item designed to assess knowledge of giftedness, support for special gifted programs/provisions, perceptions of non-elitism and academic impact; two items designed to assess contact with gifted persons; three items designed to assess power distance orientation and cognitive attitude; four items designed to assess affective attitude), the confirmatory factor analysis solution that retained the expected twelve factors included one factor (i.e., affective attitude) that was assessed with only two items, which is below the recommended “minimum of three items per factor, preferably four” items per factor (Hair et al. 2019, p. 666). Furthermore, the Comparative Fit Index (CFI) and Tucker-Lewis Index (TLI) values of this solution were below .90 (i.e., CFI = .86 and TLI = .85). The outcome was suggestive of the need to re-assess and re-conceptualize the constructs of interest to the study. For this reason, along with the adaptations that were made to some of the original scales in the survey instrument to suit the study context, exploratory factor analysis (which removes the a priori constraints of confirmatory factor analysis) was conducted.

All survey items that were previously submitted to confirmatory factor analysis were submitted to exploratory factor analysis. Using IBM SPSS Statistics version 28, and the maximum likelihood method of estimation with oblique rotation, a robust factor solution was arrived at after the progressive removal of items demonstrating low communality values (i.e., below .30), items loading onto weak factors comprising less than three items, items loading onto factors that were not theoretically interpretable, items with substantial cross-loading (i.e., loadings above .30 on more than one factor), and items with a lack of substantial loading on any factor (i.e., no loadings above .30). Hair et al. (2019) considers a factor loading of .30 to meet the minimal level for interpretability, particularly with sample sizes greater than 300.

#### 3.3.2. Latent Profile Analysis

Among the factors extracted, those relating to parental attitudes were used as indicator variables in latent profile analysis. Latent profile analysis is an analytical procedure that, unlike variable-centered procedures such as multiple regression analysis, recognizes the heterogeneity of the population from which a sample is drawn, and identifies groups of participants who display similarities in the characteristics of interest (i.e., similar types of attitudes toward gifted students and gifted education, in the case of this study; Morin et al. 2016; Wang and Wang 2020). Under this procedure, individual participants are assigned probabilities of membership into each potential latent profile (i.e., posterior probabilities) according to their responses to indicator variables, and thereafter classified into one latent profile in which he/she is estimated to have the greatest likelihood of membership (Morin et al. 2016; Wang and Wang 2020). 

The typical steps that are followed in latent profile analysis are (a) the determination of the optimal number of latent profiles, (b) the examination of the latent profile classification accuracy results, and (c) the assignment of appropriate labels to each latent profile (Wang and Wang 2020). To determine the optimal number of latent profiles, a number of models of latent profiles with different numbers of latent profiles were estimated and compared (Wang and Wang 2020). It is common practice for these models to be assessed using a combination of model fit indices and statistical tests, including the Akaike information criteria (AIC; Akaike 1983), the Bayesian information criteria (BIC; Schwarz 1978), sample-size adjusted BIC (ABIC; Sclove 1987), the Vuong-Lo-Mendell-Rubin likelihood ratio test (VLMR LRT; Lo et al. 2001), the adjusted Lo-Mendell-Rubin likelihood ratio test (aLMR LRT), and the bootstrapped likelihood ratio test (BLRT; McLachlan 1987). Other commonly considered criteria in the determination of the optimal number of latent profiles include the adequacy of the size of each latent profile (i.e., latent profiles comprising a very small number of members are meaningless), the uniqueness of each latent profile, the accuracy of the participant classifications (i.e., assessed using the probability of correct profile membership assignment or entropy values; Celeux and Soromenho 1996; Morin et al. 2011; Nagin 2005), and the theoretical interpretability of the latent profile solutions (Morin et al. 2011; Wang and Wang 2020).

In this study, the above analysis was performed using M*plus* version 8.8 with the robust maximum likelihood estimator. The number of initial stage random starts was 1000 and the number of final stage optimizations was set at 250 (i.e., 250 replications) to avoid local optimization of the maximum likelihood results. For the optimization algorithm, the exponential moving average algorithm (EMA) was used.

After making a decision on the optimal number of latent profiles, the finalized set of latent profiles was carefully examined to arrive at appropriate labels for each latent profile. Thereafter, to allow for the assessment of any predictors of the latent profile subgroups, an approach known as the classical three-step method (cf. Wang and Wang 2020) was employed. The three steps in this method are (a) the conduct of the latent profile analysis and the classification of observations on the basis of their most likely posterior probabilities, (b) the saving of the latent profile variable as an observed categorical variable (i.e., latent profile membership), and (c) the use of the saved categorical variable as an outcome variable in analysis involving predictor variables.

## 4. Results

### 4.1. Exploratory Factor Analysis

An exploratory factor solution that was free of any problematic issues was arrived at after the progressive removal of 17 items (i.e., one item designed to assess knowledge of giftedness, socio-emotional impact, academic impact, cognitive attitude and behavioral attitude; two items designed to assess contact with gifted persons; three items designed to assess perceptions of non-elitism and affective attitude; four items designed to assess support for special gifted programs/provisions). The Kaiser-Meyer-Olkin (KMO) measure of sampling adequacy (.88) and the results of the Bartlett’s test of sphericity (11,419.93, *p* < .01) of this solution indicated that factor analysis was an appropriate form of analysis on the data (Field 2018; Hair et al. 2019). The solution comprised 11 factors, according to both Kaiser’s criterion and the scree plot criterion, that were labeled as (a) Perceptions of Non-Elitism (i.e., perceptions that gifted programs/provisions are not elitist; eigenvalue = 1.12), (b) Subservience (i.e., perceptions that people at lower levels in organizations should be subservient; eigenvalue = 1.21), (c) Support for Special Gifted Education Settings (i.e., support for special educational settings to address the needs of gifted students; eigenvalue = 1.39), (d) Contact with Gifted Persons (i.e., contact with gifted people; eigenvalue = 1.73), (e) Academic Impact (i.e., positive academic impacts of gifted education; eigenvalue = 1.83), (f) Authority (i.e., recognition of the authority of people at higher levels in organizations; eigenvalue = 2.12), (g) Knowledge of Giftedness (i.e., perceptions that one is knowledgeable about giftedness; eigenvalue = 2.94), (h) School Administrative Support (i.e., support of the school administration/leadership for gifted education; eigenvalue = 3.97), (i) Self-Perceptions of Giftedness (i.e., perceptions that one is gifted; eigenvalue = 4.75), (j) Socio-Emotional Impact (i.e., positive socio-emotional impacts of gifted education; eigenvalue = 7.03), and (k) Support for Gifted Education Adaptations (i.e., support for educational adaptations to address the needs of gifted students; eigenvalue = 10.12). Collectively, these factors accounted for 66% of total variance in the data. Table 2 outlines the items that comprised each of the 11 factors, their respective factor loadings, and the Cronbach alpha values of each of these factors, while Table 3 outlines the correlations between these factors.

The major differences between this factor solution and the confirmatory factor solution (comprising the expected 12 factors) were the extraction of two factors from the items designed to assess power distance orientation (i.e., Subservience and Authority), and the merging of the items designed to assess four of the attitude factors (i.e., attitude factors other than perceptions of non-elitism) into two factors (i.e., Support for Special Gifted Education Settings and Support for Gifted Education Adaptations), in this factor solution. We note that all factors that would not have been extracted with confirmatory factor analysis, were not only theoretically interpretable, but also had sound empirical support. One of these factors, Support for Gifted Education Adaptations, which drew items designed to assess all attitude factors other than perceptions of non-elitism, had particularly strong empirical support (i.e., eigenvalue = 10.12, Cronbach α = .92, and factor loadings ranging from .49 to .87, with most factor loadings greater than .50).

In the end, the exploratory factor analysis allowed for the extraction of three attitude factors (i.e., Support for Gifted Education Adaptations, Support for Special Gifted Education Settings, and Perceptions of Non-Elitism) and eight potential predictors of these attitudes (i.e., Socio-Emotional Impact, Self-Perceptions of Giftedness, School Administrative Support, Knowledge of Giftedness, Academic Impact, Contact with Gifted Persons, Authority and Subservience).

### 4.2. Latent Profile Analysis

#### 4.2.1. Determination of the Optimal Number of Latent Profiles

Among the extracted factors, the three attitude factors (i.e., Support for Gifted Education Adaptations, Support for Special Gifted Education Settings, and Perceptions of Non-Elitism) were used as indicator variables in latent profile analysis to identify subgroups of parents with distinctive attitudes toward gifted students and gifted education. As a first step, latent profile models comprising two to six subgroups were estimated to determine the optimal number of latent profiles for the data. An examination of various model fit indices and statistical tests (i.e., AIC, BIC, ABIC, VLMR LRT, aLMR LRT, and BLRT) produced mixed findings that indicated that different models may be optimal.

As such, a second key criterion in the determination of the optimal number of latent profiles, sample size adequacy, was assessed. It was noteworthy that *all* of the models except for the two-factor model comprised a latent profile subgroup comprising only five participants (*n* = 5), and all of the models except for the two- and three-profile models comprised a latent profile subgroup comprising only four participants (*n* = 4). As both of these latent profile subgroups are excessively small to be meaningful (comprising, on average, 1% of the participants), and therefore interfered with the process of determining the optimal number of latent profiles, the nine participants associated with the profiles were removed from analysis. 

When the analysis was rerun with the remaining 322 participants, the model fit indices and the statistical tests again provided results that were somewhat mixed (see Table 4). Nevertheless, the results of the VLMR LRT, aLMR LRT, and BLRT statistical tests collectively indicated that meaningful gains would be unlikely from modeling an additional latent profile to any of the three-, four-, or five-profile models. Furthermore, each of the four-, five-, and six-profile models were found to contain one or two latent profiles (of differing profile sizes) comprising less than 10% (i.e., 3% to 9%) of the participants. These findings collectively suggested that the two- and three-profile models may be more likely than any of the other models to be the optimal model.

As such, a closer examination was made of the two- and three-profile models. Both models were evaluated to comprise latent profiles that were both theoretically interpretable, and unique with respect to other latent profiles in the respective models. Nevertheless, a key difference between the two- and three-profile models was that one large latent profile in the two-profile model, comprising greater than 70% of the participants, was essentially split into two distinct latent profiles in the three-profile model. 

Thereafter, an assessment was made of the accuracy of the classifications of the participants into the latent profiles in the two models. First, the entropy value of the three-profile model (i.e., .70) was found to be greater than the entropy value of the two-profile model (i.e., .67; see Table 4), to suggest that the retention of an additional latent profile in the three-profile model not only contributed an additional meaningful latent profile, but also improved the accuracy of the classification results. Secondly, when the classification probabilities for the most likely latent profile membership were examined for the three-profile model (see Table 5), the sound classification accuracy of the model was confirmed. Specifically, the diagonal values of the matrix in Table 5 (i.e., the correct profile membership assignment) ranged from .84 to .89 (which were above the cut-off of .70 proposed by Nagin 2005), while the off-diagonal values (i.e., the measurement errors of latent profile variables) ranged from .00 to .13.

All things considered, the latent profile model comprising three latent profiles was determined to be the optimal model for the data.

#### 4.2.2. Interpretation of the Three-Profile Model

**Figure 1:**
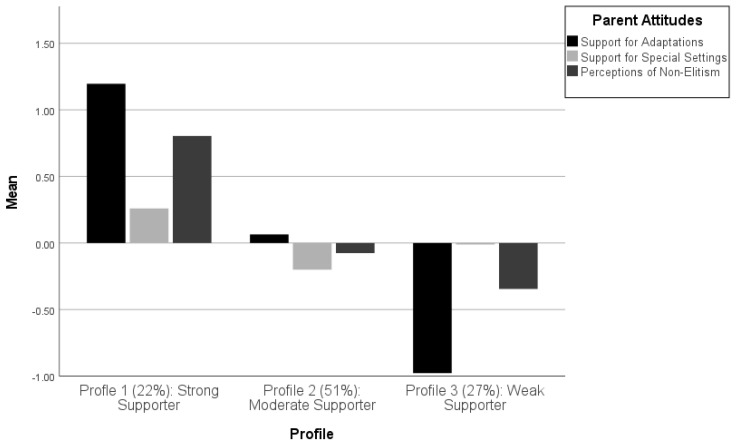
Latent profile analysis results: Three-profile model of parental attitudes toward gifted students and gifted education (Note: the values on the y-axis are standardized factor scores with a mean of zero and a standard deviation of one).

Figure 1 presents a graphical representation of the three latent profiles that comprised the optimal model, according to the mean values of the three indicator variables (i.e., Support for Gifted Education Adaptations, Support for Special Gifted Education Settings, and Perceptions of Non-Elitism) for each latent profile. The participants who were members of Profile 1 (i.e., 22% of the participants) appeared to be strongly supportive of gifted students and gifted education, as demonstrated by the largest and most positive mean values on all three indicator variables. As such, the profile was labeled “Strong Supporter of Gifted Students/Gifted Education”. In comparison, Profile 2 (51%), which comprised the largest number of participants, showed somewhat lukewarm (i.e., neither positive nor negative) attitudes toward gifted students and gifted education, and was labeled “Moderate Supporter of Gifted Students/Gifted Education”. Finally, Profile 3 (27%) was labeled “Weak Supporter of Gifted Students/Gifted Education”, as it was the only profile with negative mean scores on all three indicator variables, including the most negative scores for both Support for Gifted Education Adaptations and Perceptions of Non-Elitism.

The omnibus between-group one-way ANOVA tests demonstrated that the three profiles had statistically significant differences in the mean values of the three indicator variables (i.e., F[2, 319] = 728.95; *p* < .001 for Support for Gifted Education Adaptations; F[2, 319] = 11.99; *p* < .001 for Support for Special Gifted Education Settings; and F[2, 319] = 59.62; *p* < .001 for Perceptions of Non-Elitism). Nevertheless, Figure 1 highlights that the most pronounced differences between the profiles existed for Support for Gifted Education Adaptations, and that differences between the profiles for Perceptions of Non-Elitism and Support for Special Gifted Education Settings were substantially smaller. This was also reflected in the size of the respective F values, along with the post hoc profile difference tests with the Bonferroni correction, which were statistically significant at the *p* < .05 level for all paired profile comparisons (the one exception was for the Profile 2 vs. Profile 3 comparison for Support for Special Gifted Education Settings; *p* = .10). Collectively, these findings provided evidence that Support for Gifted Education Adaptations was the key differentiator of participant membership across the three latent profiles.

#### 4.2.3. Sociodemographic and Related Predictors of Latent Profile Group Membership

After the identification of the optimal latent profile model, a series of analyses were performed to identify statistically significant predictors of parental attitudes toward gifted students and gifted education. First of all, five potential sociodemographic and related predictors—gender, urban vs. rural locality, educational attainment, ethnicity, and perception of one’s child as gifted—were assessed. In recognition of the fact that these predictor variables are categorical variables and the latent profile analysis outcome variable is an unordered categorical variable, chi-square tests of independence were performed (see Table 6).

Of the five predictor variables, parental perception of one’s child as gifted [χ²(3) = 36.49; *p* < .001; Cramer’s V = .34], educational attainment [χ²(3) = 13.17; *p* = .010; Cramer’s V = .20], and urban vs. rural locality [χ²(3) = 6.52; *p* = .04; Cramer’s V = .14] were identified to be statistically significantly associated with latent profile membership, while gender and ethnicity were not so identified. Among the three statistically significant predictor variables, the strongest association was found for parental perceptions of the giftedness of their child, with those parents who perceived their children to be gifted more likely to have supportive attitudes. That is, among participants who considered their children to be gifted, 33% belonged to Profile 1 (“Strong Supporter of Gifted Students/Gifted Education”), 49% belonged to Profile 2 (“Moderate Supporter of Gifted Students/Gifted Education”), and 18% belonged to Profile 3 (“Weak Supporter of Gifted Students/Gifted Education”). The reverse pattern was found among participants who did not consider their children to be gifted (i.e., 7% in Profile 1, 54% in Profile 2, and 39% in Profile 3). The Cramer’s V value of .34 (*p* < .001) indicated a “medium” effect size (cf. Cohen 1988).

With respect to parental educational attainment, an overall tendency for more educated parents to have positive attitudes toward gifted students and gifted education was identified. Specifically, there was a greater representation of parents with advanced degrees (29%) in comparison to parents without university degrees (16%) in the profile with the most supportive attitudes toward gifted students/gifted education (i.e., Profile 1). The reverse pattern was found in the profile with the least supportive attitudes toward gifted students/gifted education (i.e., Profile 3). That is, only 18% of participants with advanced degrees, but 41% of participants with no university degree, were represented in this profile. The size of this effect was “small-medium” (Cramer’s V value of .20, *p* < .05; cf. Cohen 1988).

Finally, a “small” effect was found for the association between urban vs. rural locality and latent profile membership. Generally, parents living in rural areas appeared more likely to have supportive attitudes toward gifted students and gifted education than parents living in urban areas. Indeed, 35% of the rural parents were classified into Profile 1, while only 20% of the urban parents were classified into this profile.

#### 4.2.4. Psychological and Perceptual Predictors of Latent Profile Group Membership

An assessment followed of the eight psychological/perceptual variables that were extracted during exploratory factor analysis, using a series of between-group one-way ANOVA tests, for their possible prediction of latent profile membership. All post hoc pairwise profile comparisons for each ANOVA test were conducted with the Bonferroni correction.

Table 7 presents the outcomes of the analysis. All but two psychological/perceptual variables (i.e., Contact with Gifted Persons and Authority) were found to demonstrate statistically significant mean differences across the three latent profiles according to the omnibus F tests. Among the statistically significant variables, Socio-Emotional Impact [F(2, 319) = 68.22; *p* < .0001; *η*^2^ = .30] was identified to have the greatest differences in mean values across the three latent profiles. Furthermore, all pairwise group comparisons in the post hoc tests suggested highly significant group differences (*p* < .001). This “large” effect (Cohen 1988) suggested that those participants who recognize the positive socio-emotional impacts of gifted education may be more likely than those who do not recognize such impacts, to have supportive attitudes toward gifted students and gifted education.

In comparison, “medium-large” effect sizes were identified for both Academic Impact [F(2, 319) = 19.00; *p* < .0001; *η*^2^ = .11] and Knowledge of Giftedness [F(2, 319) = 16.14; *p* < .0001; *η*^2^ = .09]. In the case of Academic Impact, all post hoc pairwise profile comparisons indicated statistically significant differences in mean values across the three latent profiles, while for Knowledge of Giftedness, two out of the three post hoc pairwise profile comparisons indicated statistically significant differences in mean values across the latent profiles (the exception was Profile 2 vs. Profile 3). The findings collectively indicated that participants who recognize the positive academic impacts of gifted education, or perceive themselves to be knowledgeable about giftedness, were more likely than those participants who do not have such perceptions, to have supportive attitudes toward gifted students and gifted education.

The remaining three statistically significant predictor variables—School Administrative Support [F(2, 319) = 8.830; *p* < .0001; *η*^2^ = .05], Self-Perceptions of Giftedness [F(2, 319) = 3.949; *p* = .020; *η*^2^ = .02] and Subservience [F(2, 319) = 3.534; *p* = .030; *η*^2^ = .02]—showed “small-medium” effects. For these predictor variables, only one or two of the post hoc pairwise profile comparisons indicated statistically significant differences in mean values across the latent profiles. These findings indicated that those participants who perceive themselves to be gifted or do *not* believe that people at lower levels in organizations should be subservient may be more supportive of gifted students and gifted education. Moreover, they suggested that those participants who believe that the school administration/leadership is supportive of gifted education may be moderately supportive themselves.

#### 4.2.5. Relative Importance of Predictors

As all of the above analyses are a series of bivariate analyses between *individual* predictor variables and parental attitudes, multinomial logistic regression (that simultaneously incorporates multiple predictor variables in the one set of analyses) was performed to establish the relative importance of each predictor variable while controlling for other predictor variables. Table 8 outlines the outcomes of the multinomial logistic regression analyses by the provision of indices on the effects of each predictor variable on the likelihood of participant membership in one latent profile relative to another. For example, the first row (“female”) in Table 8 under the sub-heading “Profile 1 vs. Profile 2” presents results relating to the effects of female gender on likely membership in Profile 1 relative to Profile 2.

Five of the predictor variables were identified to be statistically significant predictors of membership into Profile 1 (“Strong Supporter of Gifted Students/Gifted Education”) relative to Profile 2 (“Moderate Supporter of Gifted Students/Gifted Education”). Among these predictors, Socio-Emotional Impact was the strongest predictor of membership between the two profiles, as indicated by the size of its odds ratio (i.e., 4.48, indicating that those who scored one unit higher on Socio-Emotional Impact were 4.48 times more likely to be classified in Profile 1 relative to Profile 2). This predictor variable was followed by the perception that one’s child is gifted (i.e., odds ratio = 4.10), Academic Impact (i.e., 2.22), Knowledge of Giftedness (i.e., 2.20), and urban vs. rural locality (i.e., 0.26).

A slightly different picture emerged for the prediction of membership into Profile 2 (“Moderate Supporter of Gifted Students/Gifted Education”) relative to Profile 3 (“Weak Supporter of Gifted Students/Gifted Education”). Six statistically significant predictors were identified. The order of importance of these variables in the prediction of profile membership, as determined by their respective odds ratios, were: perception that one’s child is gifted (i.e., 4.65), Academic Impact (i.e., 2.50), Socio-Emotional Impact (i.e., 2.29), School Administrative Support (i.e., 2.12), Self-Perceptions of Giftedness (i.e., 0.53) and no university degree vs. advanced degree (i.e., 0.31).

Slightly different again were the statistically significant predictors of membership into Profile 1 (“Strong Supporter of Gifted Students/Gifted Education”) relative to Profile 3 (“Weak Supporter of Gifted Students/Gifted Education”). Again, six statistically significant predictors were identified. The ranking of these predictor variables in the prediction of profile membership, as indicated by their odds ratios, were: perception that one’s child is gifted (i.e., 19.02), Socio-Emotional Impact (i.e., 10.27), Academic Impact (i.e., 5.56), Knowledge of Giftedness (i.e., 3.24), Self-Perceptions of Giftedness (i.e., 0.41), and urban vs. rural locality (i.e., 0.22).

Collectively, the findings of the multinomial logistic regression analyses indicated that of all predictor variables, eight variables were statistically significant predictors in at least one of the three pairwise profile comparisons. Three of these variables (i.e., perception that one’s child is gifted, Academic Impact, and Socio-Emotional Impact) were statistically significant predictors in all three profile comparisons.

## 5. Discussion

The findings of the study provide new insights into an area in which there is a relative lack of knowledge and understanding—the attitudes of parents toward gifted students and gifted education, and the factors that may predict such attitudes. Unfortunately, most of the available studies in the literature relate to the attitudes of teachers, who are likely to have different motivations, interests and perspectives, as well as experiences and education, to the parent body. This study therefore represents the first known study to offer a comprehensive and sophisticated understanding of the attitudes focused on parents—who, arguably, qualify as one of the most important stakeholder groups in the education of gifted students. Indeed, Gagné (2009, 2021) recognizes parents to be a key environmental factor that influences whether gifted students achieve to their potential.

First of all, the findings provide fresh insights into the specific types of attitudes that parents may have toward gifted students and gifted education. These attitudes—support for gifted education adaptations, support for special gifted education settings and perceptions of non-elitism—largely do *not* align with the tricomponent view of attitudes espoused in the fields of social psychology and special education (Ewing et al. 2018; Oskamp and Schultz 2005). Instead, they appear closer to the conceptualization (i.e., supportive attitudes vs. perceptions of elitism) identified among teachers in the field of gifted education (Jung 2014; McCoach and Siegle 2007; Mullen and Jung 2019; Palacios Gonzalez and Jung 2021). Nevertheless, unlike for teachers, the parent body appears to differentiate supportive attitudes for two broad categories of gifted education provisions (i.e., “moderate” provisions involving adaptations to existing provisions such as curriculum differentiation in the regular classroom, and the more “extreme” offerings within special settings such as separate gifted classes, gifted schools and possibly acceleration). The distinction between two categories of gifted education provisions in parental attitudes may be a reflection of how the parent cohort may perceive gifted education provisions (i.e., either adaptations or special settings). Furthermore, it may reflect an underlying “structure” of parental thinking with respect to gifted students/gifted education, that has a focus on the type of gifted education provisions over other possible attitude components such as affect or behavior.

Among the three components of parental attitude, support for gifted education adaptations was identified to be the most important in determining which of the three attitude profiles that parents were likely to become a member of. That is, the parent body was found to diverge most substantially in their support or non-support for gifted education adaptations. Among the other two attitude components, some variation was also observed in perceptions of non-elitism, while minimal variation was observed for support for special gifted education settings. As such, any attempts to differentiate the parent body between the three groupings identified in this study, for example to appropriately pitch any gifted education information sessions, may benefit from assessments of their attitudes toward adapting the regular curriculum for gifted students.

The latent profile model that was finally accepted ultimately provided empirical support for the mutual co-existence of three cohorts of parents with distinct attitudes toward gifted students and gifted education. The identification of more than one cohort was perhaps not unexpected, given common societal perceptions about the existence of contrasting attitudes (e.g., opposition/apathy/ambivalence vs. admiration/support) toward gifted students and gifted education (Jung 2014; Geake and Gross 2008; Lassig 2009; McCoach and Siegle 2007; Troxclair 2013). Nevertheless, this study allowed for a more nuanced understanding of these attitudes by the quantification, for the first time, of both the number of distinct cohorts that may exist and the relative sizes of these cohorts. Of particular note was the large size of the “moderate supporter” cohort (i.e., comprising more than half of the participants), who have not always been highlighted in studies to date, and the similar sizes of the cohorts that have either strongly supportive or strongly non-supportive attitudes (i.e., 22% and 27%). As such, rather than comprising two large and opposing “camps” of parents, the findings of this study suggest that neither camp is particularly large nor dominant, and that many in the parent body in fact have moderate attitudes and belong to neither of these camps. The possibility exists that the large size of the moderate supporter cohort reflects the co-existence of multiple values that are espoused in the Christian faith-based school system, that may either be conducive to supportive attitudes (e.g., a commitment to learning and the fulfillment of one’s potential) or non-supportive attitudes (e.g., inclusivity and support for social justice).

### Key Predictors of Parental Attitudes

With respect to the predictors of parental attitudes, the study findings identified nine variables that may potentially be related to membership in the three distinct cohorts of parents. These predictor variables may be classified into three tiers according to their relative importance (see Table 9). The first tier comprises the three predictor variables that have been identified to be statistically significant in the bivariate analyses *and* in all pairwise comparisons of the multinomial logistic regression analyses (i.e., perception of child as gifted, socio-emotional impact and academic impact), the second tier comprises the three predictor variables identified in the bivariate analyses *and* in two of the pairwise comparisons of the multinomial logistic regression analyses (i.e., perceived knowledge of giftedness, urban vs. rural locality and self-perceptions of giftedness), while the third tier comprises the three predictor variables that have been identified in at least the bivariate analyses (i.e., school administrative support, no university degree vs. advanced university degree, and subservience).

Regardless of this classification, the two most important predictor variables appear to be perceptions of the giftedness of one’s child and the socio-emotional impact of gifted education. The outcomes of the multinomial logistic regression analyses indeed identified these two predictors to be the top ranked predictor of parental attitudes in at least one of the three pairwise comparisons of parental attitudes. It is certainly logical and intuitive that those parents who perceive their child to be gifted will have supportive attitudes toward their child and any educational provisions that are designed to support their child’s needs (and that, in parallel, parents who do not consider their child to be gifted will not have similarly supportive attitudes). The finding is consistent with studies that have compared the attitudes of the parents of gifted students and non-gifted students. For example, Makel (2009), who compared the attitudes of parents before and after the identification of their children as gifted, noted that after identification, the parents of students who were not identified as gifted reported less favorable attitudes in comparison to the parents of students who were identified as gifted. Similarly, Wirthwein et al. (2019) noted that in comparison to the parents of non-gifted students, the parents of gifted students recognized the superior intelligence, motivation, socio-emotional adjustment, and general school functioning of their children, along with their less “problematic” nature.

The second of the most important predictor variables was socio-emotional impact, which is unsurprising in a school system that aims to educate and support the “whole person”. The identification of this predictor variable appears to reflect concerns relating to the socio-emotional issues that members of the parent body may have about gifted students and gifted education. These concerns are acknowledged in common perceptions and stereotypes about gifted students such as their greater general vulnerability to social adjustment, emotional instability and tendencies for unhealthy perfectionism (Baudson and Preckel 2013; Callahan 2017; Wiley and Hebert 2014; Wirthwein et al. 2019). Related concerns have also been noted in perceptions about the consequences of some gifted education provisions (e.g., acceleration), including social isolation (Hoogeveen 2015; Mullen and Jung 2019; Rambo and McCoach 2012). In contrast to such concerns is the recognition by other parents of the positive characteristics of giftedness including superior social competence, adaptability to new circumstances, and success in educational and career outcomes (i.e., harmony hypothesis; Preckel et al. 2015; Wirthwein et al. 2019). Many such parents also recognize the potential benefits of gifted education provisions in fostering friendships and a sense of belonging with other gifted students, along with the potential dangers of retaining gifted students in the regular classroom that may lack important social opportunities (Vidergor and Azar Gordon 2015). It naturally follows that those parents in this study who have concerns about the possible negative socio-emotional impacts of giftedness/gifted education may have non-supportive attitudes, while those who recognize the positive socio-emotional impacts may have highly supportive attitudes.

The last of the top tier predictors identified in this study was academic impact. While there appears to be a general recognition of the high academic potential of gifted students (Baudson and Preckel 2013; Preckel et al. 2015), some differences may exist among the parent body in their attitudes toward gifted education provisions. On the one hand, there are beliefs that gifted education provisions are necessary to allow gifted students to achieve to their potential, provide the necessary challenge and intellectual stimulation, address the boredom that these students may otherwise experience, and prevent underachievement (Morawska and Sanders 2009; Vidergor and Azar Gordon 2015). On the other hand, some parents appear to have concerns relating to the potential for academic burnout, lower academic results than would have been possible under a regular curriculum due to the increased difficulty of content, and possible gaps in learning due to a need to miss regular classwork (Rambo and McCoach 2012; Vidergor and Azar Gordon 2015). Alternatively, the concerns of parents may relate not to the offering of gifted education provisions, but rather to the anticipated low quality and limited number of such provisions that may typically be available in schools (Hertzog and Bennett 2004; Huff et al. 2005). Therefore, those parents in this study who recognize the positive academic impacts of gifted education are likely to have supportive attitudes, while those who have concerns about the possible negative academic impacts of gifted education may be unsupportive.

In comparison to the above-mentioned predictors, the variables that do not form part of the top tier of predictor variables—perceived knowledge of giftedness, urban vs. rural locality, self-perceptions of giftedness, school administrative support, educational attainment and subservience—appear likely to have a less substantial and less systematic impact on parent attitudes toward gifted students and gifted education.

## 6. Implications

The findings of the study have a number of implications for both research and practice.

### 6.1. Implications for Research

First of all, in terms of research, it may be useful to replicate the study in school systems affiliated with different faiths, and in school systems that are not faith-based (i.e., schools in the public and private sectors) in Australia, in other cultural contexts, and in other countries. Such replications may reveal interesting similarities and differences in the profiles of parental attitudes, the sizes of the groups with different attitude profiles, and the significant predictors of these attitude profiles in different school systems. Relatedly, future studies could introduce some variations to the data collected in this study. For example, in recognition of the fact that two of the three extracted attitude factors related to attitudes toward gifted education provisions, data could be collected to assess attitudes toward other gifted education provisions, such as mentorships and counseling, to assess whether they may lead to the extraction of additional factors. Furthermore, it may be useful to investigate the relevance of predictor variables that were not investigated in this study, but may nevertheless have the potential to influence parental attitudes (e.g., cultural variables other than power distance orientation, variables related to politics such as political orientation, and religious affiliation). Moreover, in recognition of the quantitative nature of many studies on attitudes toward giftedness and gifted education, future studies could adopt a qualitative design that may not only provide greater scope for non-anticipated findings, but also potentially deeper and richer understandings of parental attitudes toward gifted students and gifted education. Reflecting a common focus in Australian educational contexts on attending to the needs of disadvantaged student groups (e.g., low socio-economic status, Indigenous, rural, and special needs students; Education Council 2019; Lamb et al. 2020), which also aligns with the values of the participating school system on inclusivity and social justice, one such study could investigate any perceived conflicts between the provision of support for disadvantaged students and gifted students, in the formation of parental attitudes toward gifted students and gifted education.

The many findings of this study also raise a number of questions that may be conducive to further research. Specifically, the extraction of separate factors for attitudes toward gifted education adaptations and special gifted education settings lead to questions about the precise “borderline” between these two gifted education provisions. That is, while curriculum differentiation in the regular classroom may be seen as a gifted education adaptation, and special schools for gifted students may be seen as a special setting for gifted students, where specifically do acceleration provisions such dual enrolment (i.e., simultaneous enrollment in different schools; Assouline et al. 2015) lie? Also, why is there substantial variation between parents in their attitudes toward gifted education adaptations, but minimal variation in their attitudes toward special gifted education settings? Other questions relate to the view, often held by critics of gifted education, that gifted education provisions may lead to the segregation of students by race, socio-economic status and other factors related to differences in economic/political/cultural capital. As the study findings indicate that ethnicity and educational attainment may *not* be strong predictors of parental attitudes toward gifted students and gifted education, questions arise as to whether the findings may be different if alternative measures of race and socio-economic status (e.g., non-self-reported measures of race and parental income) were used, along with the cross-contextual/cross-cultural/cross-national applicability of the view that gifted education may be lead to the segregation of students.

### 6.2. Implications for Practice

Along with implications for research, the study findings provide a number of implications for practice. First of all, there appears to be a clear need for regular and effective parent education or information sessions on giftedness and gifted education, to promote a proper understanding of the phenomenon, and to discourage a reliance on personal impressions, stereotypes or myths among the parent body. Furthermore, to ensure that such education and information sessions are appropriately pitched to individual parents, it may be useful to introduce processes that identify the different groups of parents with distinct attitude profiles, and for any education/information sessions targeted to each group to be appropriately modified for each group. Possibly, these identification processes may benefit from the use of identification instruments that are based on the scales used in this study relating to parental attitudes toward gifted education adaptations, as attitudes toward gifted education adaptations have proven to be a key differentiator of parental attitudes.

The findings of the study also provide some suggestions on ways to promote supportive attitudes among parents toward giftedness and gifted education. Specifically, they suggest that communications with the parent body could focus on highlighting the positive socio-emotional and academic impacts of gifted education, such as the opportunity to foster strong friendships with like-ability and like-minded students, and the opportunity to achieve to one’s potential in an intellectually stimulating environment (Morawska and Sanders 2009; Vidergor and Azar Gordon 2015). They also suggest that any parent education and information sessions may be conducive to positive parent attitudes, as such sessions may eventually lead to a greater awareness of giftedness among their own children.

It must be noted that positive parental attitudes toward gifted students and gifted education may not always lead to positive outcomes for gifted students. Nevertheless, positive parental attitudes may be conducive to parent actions, such as advocacy efforts, to support the educational and socio-emotional needs of their gifted children, which may eventually lead to positive student outcomes (Duquette et al. 2011; Mun et al. 2021). Parent advocacy is often motivated by a need to intervene in the education of gifted children, and may take place in the home (e.g., establishment of academic and related expectations), the school (e.g., involvement in school governance, the organization of gifted programs, and the development of school policy), and at state and national levels (e.g., lobby of relevant government departments and contribution to state/national policy formulation), often in collaboration with other parents and other key stakeholders in the education of gifted students (Duquette et al. 2011; Mun et al. 2021).

## 7. Limitations

A number of study limitations need to be acknowledged to aid reader interpretations. First, the study findings relate to analyses of data collected from the parents of students enrolled in a single K–12 Christian faith-based school system in Australia. As such, caution needs to be exercised before the findings are generalized to other school systems in Australia, or to school systems in other cultural contexts and countries. Second, as participation in the study was voluntary, the participants may on average have had more favorable attitudes toward gifted students and gifted education than parents in the school system generally. As such, the size of the parent profile with the least supportive attitudes may be an underestimation. Finally, it is possible that the findings of the study may reflect a single source bias, as the collected data were not corroborated with data from other parties. If the study was designed such that both parents of each child enrolled in the school system were asked to participate, a more valid and reliable picture of parent attitudes toward gifted students and gifted education may have been possible.

## Figures and Tables

**Table 1:** Survey instrument.

Variable	Details	Scale Origin	Cronbach α	Sample Item	Response Format
Sociodemographic Variables	Age, Gender, Educational attainment, Place of birth, Ethnicity, Urban/rural locality (1 item each)			“Gender”	Open-ended
Child-related variables	Child’s school, Giftedness of child (1 item each)			“Do you consider your child to be gifted?”	Open-ended
Traditional attitudes toward gifted students/ gifted education	Supportive attitudes (6 items)	Originally developed by McCoach and Siegle (2007), and slightly modified by Jung (2014)	.76 (McCoach and Siegle 2007);	“Our schools should offer special educational services for gifted students”	7-point Likert-type scale (*strongly agree* to *strongly disagree*)
			.72 (Jung 2014)		
	Perceptions of elitism (6 items)	Originally developed by McCoach and Siegle (2007), and slightly modified by Jung (2014)	.80 (McCoach and Siegle 2007);	“Special programs for gifted students have the drawback of creating elitism”	7-point Likert-type scale
			.75 (Jung 2014)		
Tricomponent view of attitudes	Cognitive attitude (6 items)	Mahat (2008), which had a special education context	.77 (Mahat 2008)	“I believe that gifted students should be taught in special settings for gifted students” modified from “I believe that students with a disability should be taught in special education schools”	7-point Likert-type scale
	Affective attitude (6 items)	Mahat (2008), which had a special education context	.78 (Mahat 2008)	“I get irritated when I am unable to understand gifted students” modified from “I get irritated when I am unable to understand students with a disability.”	7-point Likert-type scale
	Behavioral attitude (6 items)	Mahat (2008), which had a special education context	.91 (Mahat 2008)	“I am willing to encourage gifted students to participate in social activities” modified from “I am willing to encourage students with a disability to participate in all social activities in the regular classroom”	7-point Likert-type scale
Psychological/ perceptual predictors	Power distance orientation (7 items)	“Relational hierarchy” scale in Maznevski et al. (1997), slightly modified by Jung (2014)	.74 (Jung 2014)	“People at lower levels in organizations should not have much authority”	7-point Likert-type scale
	Perceived knowledge of giftedness (7 items)	Jung (2014) from scales including Renzulli’s (1975) Key Features Model for evaluation of gifted programs and the subjective knowledge scale (Flynn et al. 2000)	.86 (Jung 2014);	“I know quite a lot about giftedness”	7-point Likert-type scale
			.91 (Mullen and Jung 2019)		
	Contact with gifted persons (7 items)	Jung (2014) from scales including Sigleman and Welch’s (1993) survey of interracial contact, Koenig et al.’s (1993) Duke Social Support Index, and Lechler’s (2001) social interaction scales	.77 (Jung 2014);	“I know a gifted person who I would consider to be a close personal friend”	7-point Likert-type scale
			.80 (Mullen and Jung 2019)		
	Self-perceptions of giftedness (6 items)	McCoach and Siegle (2007; 5 items) and Jung (2014; 1 item)	.94 (McCoach and Siegle 2007);	“I am gifted”	7-point Likert-type scale
			.88 (Jung 2014);		
			.95 (Mullen and Jung 2019)		
	School administrative support (6 items)	Palacios Gonzalez and Jung (2021), which was adapted from Rambo and McCoach (2012). Both scales had a focus on acceleration	.73 (Palacios Gonzalez and Jung 2021)	“My child’s school principal would be open to special programs/provisions for gifted students” modified from “My principal would be open to considering acceleration for a gifted student”	7-point Likert-type scale
	Socio-emotional impact (6 items)	Palacios Gonzalez and Jung (2021), which was adapted from Rambo and McCoach (2012). Both scales had a focus on acceleration	.73 (Palacios Gonzalez and Jung 2021)	“Gifted programs/ provisions are harmful to a student’s emotional well- being” modified from “Acceleration is harmful to a student’s emotional well-being”	7-point Likert-type scale
	Academic impact (6 items)	Academic benefits scale in Rambo and McCoach (2012), which had a focus on acceleration	.75 (Rambo and McCoach 2012)	“Gifted students placed in special gifted programs are likely to feel confident about their academic abilities” modified from “Students who accelerate in a specific subject are more likely to feel confident in that subject than students of the same ability who did not accelerate”	7-point Likert-type scale

**Table 2:** Factor solution.

Factors/Items	Loading	Alpha
Support for Gifted Education Adaptations		.92
F6. I am willing to support the adaptation of the curriculum to meet the individual needs of gifted students	.87	
F17. I am willing to support the modification of the learning environment to meet the needs of gifted students	.87	
F30. I believe that appropriate modifications should be made to the learning environment to cater to the needs of gifted students	.86	
F27. I am willing to support the adaptation of assessment practices for gifted students in order to cater to their specific needs	.83	
F16. I am willing to provide the necessary support for gifted students	.80	
F19. I believe that the regular curriculum of the school should be adapted to meet the needs of gifted students	.71	
F22. I am willing to adapt the way I interact with gifted students to address their specific needs	.68	
F20. I am uncomfortable when the needs of gifted students are not supported in the classroom	.67	
F3. Our schools should offer special educational services for gifted students	.60	
F11. I get upset when gifted students cannot progress academically due to the constraints of the curriculum	.53	
F2. I believe that gifted students should be allowed to progress academically in school at a rate that is commensurate with their ability level	.49	
F15. Gifted students need special attention to fully develop their talents	.49	
Socio-Emotional Impact		.87
E6. Gifted students who are provided with special educational services may experience academic burnout *	.84	
E2. Gifted students who are placed in special gifted programs may experience a lot of stress *	.79	
E11. Gifted programs/provisions are harmful to a student’s social wellbeing *	.79	
E5. Gifted programs/provisions are harmful to a student’s emotional wellbeing *	.75	
E12. Gifted students who are placed in special gifted programs may not participate in many social activities *	.68	
E17. Gifted students who receive special educational interventions may feel pressure to perform well *	.47	
Self-Perceptions of Giftedness		.92
D16. Most of my family and friends consider me gifted	.97	
D20. People consider me gifted	.96	
D11. I am gifted	.77	
D14. I was, or could have been, in a gifted program in school	.74	
D18. I would very much like to be considered a gifted person	.57	
School Administrative Support		.91
E10. The leadership at my child’s school is open to special educational interventions for gifted students	.92	
E1. My child’s school would consider offering special educational services for gifted students	.84	
E4. My child’s school principal would be open to special programs/provisions for gifted students	.83	
E16. The culture at my child’s school is supportive of gifted education	.80	
E9. My child’s school system would support gifted education	.75	
E15. Guidelines for gifted education may be created at my child’s school	.56	
Knowledge of Giftedness		.87
D10. I am knowledgeable about the types of classroom activities that are suitable for gifted students	.86	
D4. I understand the needs of gifted students	.80	
D8. I am familiar with some of the goals and objectives of programs designed for gifted students	.74	
D6. I know quite a lot about giftedness	.71	
D19. I have a fairly good idea about how to identify gifted people	.57	
D15. Compared to most people, I don’t know a lot about giftedness *	.52	
Authority		.78
C2. People at higher levels in organizations have a responsibility to make important decisions for people below	.75	
C1. A hierarchy of authority is the best form of organization in educational or professional settings	.73	
C3. People should be rewarded based on their level in the organization	.63	
C4. The highest ranking person in a team should take the lead	.61	
Academic Impact		.79
E13. Gifted students who are given special educational services are likely to go to university	.78	
E8. Gifted students who are offered special educational interventions are likely to be admitted into highly selective courses at university	.73	
E18. Gifted students who are given a special education are likely to pursue university studies in the area in which they received special education	.66	
E7. Gifted students placed in special gifted programs are likely to feel confident about their academic abilities	.64	
E3. Gifted students who receive special educational services are more likely to achieve better academic results than gifted students who do not receive such services	.46	
Contact with Gifted Persons		.81
D5. Many of my acquaintances are gifted	.83	
D12. I regularly come across gifted people in my day-to-day life	.66	
D2. I believe that a lot of gifted people live in my neighborhood	.61	
D13. Most of my family and friends are gifted	.59	
D3. I know a gifted person who I would consider to be a fairly close personal friend	.41	
Support for Special Gifted Education Settings		.63
F9. I believe that gifted students should be taught in special settings for gifted students	.68	
F26. I believe that gifted students should be placed in special schools so that they do not experience social rejection	.60	
F21. I am disconcerted that gifted students are required to remain in the regular classroom, regardless of their level of giftedness	.55	
Subservience		.70
C6. Organizations should have separate facilities (such as eating areas) for higher level people	.60	
C5. People at lower levels in organizations should carry out the requests of people at higher levels without question	.51	
C7. People at lower levels in organizations should not have much authority	.46	
Perceptions of Non-Elitism		.80
F28. When gifted students are put in special classes, the other students feel devalued *	.67	
F18. By separating students into gifted and non-gifted groups, we increase the labeling of students as strong-weak, good–less good, etc. *	.67	
F24. Special programs for gifted students have the drawback of creating elitism*	.51	

* reverse coded.

**Table 3:** Factor correlations.

	1	2	3	4	5	6	7	8	9	10	11
1. Support for Gifted Education Adaptations	1.00										
2. Socio-Emotional Impact	.54 **	1.00									
3. Self-Perception of Giftedness	.15 **	.10	1.00								
4. School Administrative Support	.05	−.07	−.01	1.00							
5. Knowledge of Giftedness	.31 **	.13 *	.51 **	.06	1.00						
6. Authority	.14 *	.16 **	.13 *	.05	.06	1.00					
7. Academic Impact	.38 **	.20 **	.17 **	.08	.09	.44 **	1.00				
8. Contact with Gifted Persons	.10	−.02	.62 **	.03	.45 **	.13 *	.19 **	1.00			
9. Support for Special Gifted Education Settings	.09	.16 **	.04	−.13 *	−.01	.33 **	.40 **	.18 **	1.00		
10. Subservience	−.15 **	−.21 **	.05	−.12 *	.02	.21 **	.10	.06	.12 *	1.00	
11. Perceptions of Non-Elitism	.42 **	.59 **	.08	−.02	.13 *	−.02	.04	.08	.05	−.03	1.00

* *p* < .05, ** *p* < .01.

**Table 4:** Results of model fit of five solutions from latent profile analysis.

Latent Profiles	Free Parameters	Log Likelihood	AIC	BIC	ABIC	Entropy	VLMR LRT *p*-value	aLMR LRT *p*-value	BLRT *p*-value
2	10	−1169	2358	2396	2364	.67	< .001	< .001	< .001
3	14	−1160	2349	2402	2357	.70	.087	.097	< .001
4	18	−1155	2347	2415	2358	.75	.212	.227	.286
5	22	−1148	2341	2424	2354	.76	.093	.102	< .001
6	26	−1139	2330	2428	2345	.79	.348	.360	.020

Notes. AIC = Akaike information criteria; BIC = Bayesian information criteria; ABIC = Sample-size adjusted BIC; VLMR LRT = Vuong-Lo-Mendell-Rubin likelihood ratio test; aLMR LRT = Adjusted Lo-Mendell-Rubin likelihood ratio test; BLRT = Bootstrapped likelihood ratio test; The likelihood ratio tests are conducted to compare nested models (i.e., between models with [*k* − 1] and *k* latent profiles).

**Table 5:** Three-profile model: Classification information.

Classification Probabilities for the Most Likely Latent Profile Membership (Row) by Latent Profile (Column)				Final Counts and Proportions for the Latent Profiles Based on Estimated Posterior Probabilities		
	Profile 1	Profile 2	Profile 3		Count	Proportion
Profile 1	.89	.11	.00	Profile 1	72	.22
Profile 2	.05	.84	.11	Profile 2	164	.51
Profile 3	.00	.13	.87	Profile 3	86	.27

**Table 6:** Chi-square test results of the associations between the three latent profiles and five sociodemographic and related variables.

		Gender		Urban vs. Rural		Perceive Child Gifted		Educational Attainment			Ethnicity			
		Female (*n* = 253)	Male (*n* = 67)	Urban (*n* = 263)	Rural (*n* = 54)	Yes (*n* = 187)	No (*n* = 134)	Non- University (*n* = 85)	Bachelor Degree (*n* = 167)	Advanced Degree (*n* = 66)	Anglo-Saxon (*n* = 205)	European (*n* = 55)	Asian (*n* = 33)	Other (*n* = 16)
Profile 1	Count	56	16	52	19	62	10	14	38	19	43	12	10	4
	%	22%	24%	20%	35%	33%	7%	16%	23%	29%	21%	22%	30%	25%
Profile 2	Count	133	29	139	21	92	72	36	90	35	107	30	15	9
	%	53%	43%	53%	39%	49%	54%	42%	54%	53%	52%	54%	46%	56%
Profile 3	Count	64	22	72	14	33	52	35	39	12	55	13	8	3
	%	25%	33%	27%	26%	18%	39%	41%	23%	18%	27%	24%	24%	19%
Pearson χ^2^		2.09		6.52 *		36.49 ***		13.17 *			2.08			
Cramer’s V		.08		.14 *		.34 ***		.20 *			.06			
*p* value		.35 (*ns*)		.04		< .001		.01			.91 (*ns*)			

Notes. All percentage values are percentage values within a variable (i.e., columns add to 100%); European ethnicity includes both Western and Eastern European ethnicities; Asian ethnicity includes East Asian, South Asian, and Southeast Asian ethnicities; and Other ethnicity includes Latin American, African and mixed ethnicities; *ns* = not statistically significant at the .05 level; * *p* < .05, *** *p* < .001.

**Table 7:** Group differences of the three latent profiles on key psychological/perceptual predictor variables.

Predictor	Profile	Mean	SD	F	Sig.	*η* ^2^	*p*-value (Post Hoc Test with Bonferroni Correction)	
							Profile 1	Profile 2
Knowledge of Giftedness	Profile 1	0.50	0.99	16.14 ***	< .001	.09		
	Profile 2	−0.10	0.94				< .001	
	Profile 3	−0.29	0.74				< .001	.35
Contact with Gifted Persons	Profile 1	0.17	0.94	2.46	.09	.02		
	Profile 2	−0.10	0.90				.09	
	Profile 3	−0.06	0.81				.32	1.00
Self-Perceptions of Giftedness	Profile 1	0.25	0.98	3.95 *	.02	.02		
	Profile 2	−0.11	0.99				.02	
	Profile 3	−0.08	0.80				.09	1.00
School Administrative Support	Profile 1	−0.17	1.11	8.83 ***	< .001	.05		
	Profile 2	0.20	0.90				.01	
	Profile 3	−0.26	0.76				1.00	< .001
Academic Impact	Profile 1	0.43	0.99	19.00 ***	< .001	.11		
	Profile 2	−0.03	0.77				< .001	
	Profile 3	−0.38	0.76				< .001	.004
Social-Emotional Impact	Profile 1	0.90	0.89	68.22 ***	< .001	.30		
	Profile 2	−0.06	0.75				< .001	
	Profile 3	−0.49	0.69				< .001	< .001
Authority	Profile 1	0.09	0.97	1.63	.20	.01		
	Profile 2	0.00	0.90				1.00	
	Profile 3	−0.15	0.77				.24	.55
Subservience	Profile 1	−0.19	0.76	3.53 *	.03	.02		
	Profile 2	−0.05	0.86				.64	
	Profile 3	0.15	0.70				.03	.21

Note. * *p* < .05, *** *p* < .001.

**Table 8:** Multinominal logistic regression to assess the prediction of latent profile membership.

Predictors	Profile 1 vs. Profile 2				Profile 2 vs. Profile 3				Profile 1 vs. Profile 3			
	B	S.E.	Sig.	Exp(B)	B	S.E.	Sig.	Exp(B)	B	S.E.	Sig.	Exp(B)
Female	−0.022	0.468	.963	0.979	0.588	0.404	.145	1.801	0.567	0.569	.319	1.763
Urban	−1.357 **	0.512	.008	0.257	−0.159	0.482	.742	0.853	−1.516 *	0.640	.018	0.220
Perception of child as gifted	1.410 **	0.532	.008	4.096	1.536 ***	0.406	.000	4.645	2.946 ***	0.632	< .001	19.023
No university degree	0.078	0.617	.899	1.081	−1.180 *	0.530	.026	0.307	−1.101	0.759	.147	0.332
Bachelor degree	0.286	0.479	.551	1.331	−0.347	0.479	.469	0.707	−0.061	0.637	.924	0.941
Anglo-Saxon	0.225	0.888	.800	1.253	−0.349	0.791	.659	0.705	−0.124	1.115	.912	0.884
European	0.205	0.955	.830	1.228	−0.432	0.881	.624	0.649	−0.227	1.217	.852	0.797
Asian	−0.508	1.051	.629	0.602	−0.946	0.983	.336	0.388	−1.455	1.359	.284	0.234
Knowledge of Giftedness	0.790 **	0.249	.001	2.202	0.387	0.221	.080	1.473	1.177 ***	0.313	< .001	3.244
Contact with Gifted Persons	0.017	0.284	.952	1.017	−0.156	0.269	.562	0.856	−0.139	0.367	.705	0.870
Self-Perception of Giftedness	−0.253	0.284	.373	0.777	−0.644 *	0.283	.023	0.525	−0.897 *	0.377	.017	0.408
Administrative Support	−0.390	0.205	.057	0.677	0.750 ***	0.209	< .001	2.117	0.360	0.265	.175	1.433
Academic Impact	0.799 **	0.283	.005	2.224	0.917 ***	0.252	< .001	2.502	1.716 ***	0.360	.000	5.564
Socio-Emotional Impact	1.499 ***	0.281	< .000	4.479	0.830 **	0.254	.001	2.294	2.330 ***	0.361	< .001	10.274
Authority	−0.370	0.271	.172	0.690	−0.244	0.222	.273	0.784	−0.614	0.332	.064	0.541
Subservience	0.023	0.301	.940	1.023	−0.409	0.251	.103	0.664	−0.386	0.364	.289	0.680

Notes. S.E. = standard error of the coefficient; the coefficients reflect the effects of the predictors on the likelihood of membership into the first listed profile relative to the second listed profile; the reference category of both “No university degree” and “Bachelor degree” was “Advanced degree (either Master’s or Doctoral degree)”; the reference category of Anglo-Saxon, European and Asian ethnicity was Other ethnicity; the intercepts are not included in the table; * *p* < .05, ** *p* < .10, *** *p* < .001.

**Table 9:** Three tiers of predictors of parent attitudes toward gifted students and gifted education.

Tier	Predictors
1	Perceptions of child as gifted
	Socio-emotional impact
	Academic impact
2	Knowledge of giftedness
	Rural locality
	Self-perceptions of giftedness
3	School administrative support
	Educational attainment
	Subservience

## Data Availability

The data that support the findings of this study are available on request from the corresponding author.
